# Comparative genomic analysis of *pleurotus* species reveals insights into the evolution and coniferous utilization of *Pleurotus placentodes*

**DOI:** 10.3389/fmolb.2023.1292556

**Published:** 2023-11-06

**Authors:** Lei Sun, Xiaolei Yin, Frederick Leo Sossah, Xuerong Han, Yu Li

**Affiliations:** ^1^ Jilin Province Key Laboratory of Fungal Phenomics, Jilin Agricultural University, Changchun, China; ^2^ International Cooperation Research Center of China for New Germplasm Breeding of Edible Mushrooms, Jilin Agricultural University, Changchun, China; ^3^ Council for Scientific and Industrial Research (CSIR), Oil Palm Research Institute, Coconut Research Programme, Sekondi, Ghana

**Keywords:** *Pleurotus placentodes*, genome sequencing, coniferous utilization, evolution, adaption

## Abstract

*Pleurotus placentodes* (PPL) and *Pleurotus cystidiosus* (PCY) are economically valuable species. PPL grows on conifers, while PCY grows on broad-leaved trees. To reveal the genetic mechanism behind PPL’s adaptability to conifers, we performed *de novo* genome sequencing and comparative analysis of PPL and PCY. We determined the size of the genomes for PPL and PCY to be 36.12 and 42.74 Mb, respectively, and found that they contain 10,851 and 15,673 protein-coding genes, accounting for 59.34% and 53.70% of their respective genome sizes. Evolution analysis showed PPL was closely related to *P. ostreatus* with the divergence time of 62.7 MYA, while PCY was distantly related to other *Pleurotus* species with the divergence time of 111.7 MYA. Comparative analysis of carbohydrate-active enzymes (CAZYmes) in PPL and PCY showed that the increase number of CAZYmes related to pectin and cellulose degradation (e.g., AA9, PL1) in PPL may be important for the degradation and colonization of conifers. In addition, geraniol degradation and peroxisome pathways identified by comparative genomes should be another factors for PPL’s tolerance to conifer substrate. Our research provides valuable genomes for *Pleurotus* species and sheds light on the genetic mechanism of PPL’s conifer adaptability, which could aid in breeding new *Pleurotus* varieties for coniferous utilization.

## 1 Introduction

Coniferous trees are a primary source of forest resources, generating ten million tons of conifer waste each year due to tree aging, cutting, and industrial waste. Unfortunately, most of this waste is only used as fuel, resulting in low utilization and environmental pollution (Rominiyi et al., 2017). Despite continuous research efforts to utilize these wastes by extracting effective components of turpentine and controlling soybean cyst nematodes (Silori et al., 2019), this approach still requires a significant amount of manpower and material resources. Therefore, finding an effective way to utilize these conifer wastes and promote waste reuse is crucial for environmental protection, sustainable development, and increasing people’s income.

Edible mushroom production is an important component of fully utilizing agricultural and forestry waste (Croan, 2004; Wang et al., 2021). China is the world’s largest producer of edible mushrooms, accounting for approximately 75% of the world’s yield (Liu et al., 2018), which translates to around 40 million tons of cultivation substrate consumed annually. Although traditionally, the artificial cultivation substrate of edible mushrooms has been based on broad-leaved wood sawdust rich in lignocellulose, conifers are also rich in lignocellulose but contain a large amount of turpentine acids and phenols. These compounds destroy the integrity of the cell membrane and seriously hinder the growth and development of edible mushroom mycelium and fruiting bodies, which limits the use of conifers as a substrate for producing edible mushrooms (Chen et al., 2002; Croan, 2004). In addition, the composition of coniferous and broad-leaved trees are also different and how these different compositions affect the growth of mushrooms is also still unkonwn. If coniferous tree waste can also be normally used for mushroom production, it will enormously save costs, protecting the environment, and promoting the development of the edible mushroom industry.

In 1852, the British mycologist Berkeley was first described PPL as growing on decaying wood of *Betula* (Berkeley, 1852), which breaks the idea that *Pleurotus* species are difficult to complete growth and development on the conifer substrate. After 164 years, PPL was discovered growing on *Betula* and *Picea* in the Subalpine Forests of Tibet and Yunnan, China, at altitudes of 3,000–4,200 m (Liu et al., 2016). It has since been successfully domesticated, cultivated, and verified as a non-toxic, edible, and highly nutritious mushroom (Wang et al., 2018), providing a positive strategy for the cyclic utilization of conifer waste. However, how does PPL degrade coniferous substrates composed of different types lignocellulose? As the main enzymes for lignocellulose degradation, what is the difference of CAZYmes composition between PPL and other species? What measures are contains to deal with the antibacterial substances in coniferous trees to ensure own growth? These questions have not been answered in detail at present.

Currently, the NCBI hosts 11 publicly available genomes of *Pleurotus* species, including *P*. *ostreatus*, *P*. *cornucopiae*, *P*. *platypus*, *P*. *citrinopileatus*, *P*. *floridanus*, *Pleurotus pulmonarius*, *P*. *tuoliensis*, *P*. *salmoneostramineus*, *Pleurotus eryngii*, *P*. *ostreatoroseus*, and *P*. *tuber-regium* (https://www.ncbi.nlm.nih.gov/genome/?term=pleurotus). Additionally, Fu et al. (2022) have sequenced 13 more genomes of *Pleurotus* species, thereby expanding our understanding of the genus. Among these 13 genomes, seven were sequenced for the first time, and they belong to the following species: *P*. *abieticola*, *P*. *djamor*, *P*. *eryngii* var. *ferulae*, *P*. *giganteus*, *P*. *nebrodensis*, *P*. *populinus*, and *P*. *sapidus*. While many *Pleurotus* species are known to grow on broad-leaved trees or Apiaceae plants, some can also be found on conifers. Despite several studies available on the phylogeny (Dai et al., 2019) stress response, growth and development (Xu et al., 2021a; Zhang et al., 2022), substrate-biased gene regulation (Wu et al., 2021), and lignocellulose-decay enzymes (Araújo et al., 2021) of *Pleurotus*, there is still a lack of research on their adaptability to conifers, which may be due to insufficient genomic data available for the species that grow on conifer substrates.

In this study, we aimed to provide high-quality genome assemblies of PPL and PCY, which are adapted to conifer and broadleaf substrates, respectively. Our main objectives were to compare the genomes of these two species and identify differences in their evolution and structure. Additionally, we aimed to determine the phylogenetic relationship between PPL and PCY using nuclear single-copy orthologous genes, and to investigate the molecular features of PPL’s adaptation to conifer substrates at the genome level. These reference genomes and comparative analyses will serve as important genetic resources for understanding the substrate utilization, evolution, and ecology of the *Pleurotus* genus.

## 2 Materials and methods

### 2.1 Experimental materials

The *de novo* genome sequencing were carried out with the monokaryon strains of PPL and PCY preserved in the Engineering Research Center of the Ministry of Education of Jilin Agricultural University, China.

### 2.2 Genome extraction and sample preparation

The dikaryon strains of PPL and PCY were inoculated on the potato dextrose agar (PDA) medium overlaid with cellophane sheets. The cultures were grown in a light-free environment at 25°C for 10 days. Afterward, 300 mg of hyphae was collected and transferred to Eppendorf tubes, where they were subjected to enzymatic hydrolysis using 2% lysozyme (Guangdong Institute of Microbiology, China) at 30°C for 4 h. Mononuclear hyphae were obtained by regenerating the protoplasts and staining the nucleic acid with 4′,6-diamidino-2-phenylindole (DAPI, Sigma-Aldrich, United States) before identification under an optical microscope. The mononuclear hyphae were then cultured on a PDA medium overlaid with cellophane. The mononuclear hyphae were cultured in PDA medium overlaid with cellophane for 25°C for 15 days, and 100 mg hyphae was collected separately for each strain. Finally, the highly efficient NuClean Plant Genomic DNA extraction kit (CWBIO, Beijing, China) was used to extract the genomic DNA for each strains. Genomic integrity, purity, and concentration were evaluated using 0.8% agarose gel electrophoresis, Nanodrop 2000 (Thermo Fisher Scientific, Foster City, CA, United States), and Qubit (Thermo Fisher Scientific, Foster City, CA, United States), respectively.

### 2.3 De novo genome sequencing and assembly of PPL and PCY strains

*De novo* genome sequencing of PPL and PCY was performed with a 20 k library size using a PacBio sequel platform in the Engineering Research Center of the Ministry of Education of Jilin Agricultural University, China (Wang et al., 2019a; Sossah et al., 2019). The subreads were assembled using SMARTdenovo (https://github.com/ruanjue/smartdenovo, version 1.0.0) (Liu et al., 2021). The Core Eukaryotic Genes Mapping Approach (CEGMA) (Parra et al., 2007) and Benchmarking Universal Single-Copy Orthologs (BUSCO) (Waterhouse et al., 2018) were used to test the accuracy and completeness of the assembled this two genomes. Both genome sequences have been submitted to Figshare database (https://figshare.com/projects/Genome_sequencing_of_Pleurotus_placentodes_and_Pleurotus_cystidiosus/165418).

### 2.4 Genome annotation of PPL and PCY

*De novo* and homologous prediction strategies were used to annotate the two genomes of *Pleurotus*, including the wild strain PPL from the Tibetan Plateau and the PCY strains. Four reference species, including *Agaricus bisporus*, *Coprinopsis cinerea*, *Pleurotus ostreatus,* and *Schizophyllum commune* were used for homologous prediction. Augustus (version 3.3.2), Genescan (version 3.7), GlimmerHMM (version 3.0.4), and SNAP software (semi-hmm-based nucleic acid parser) were used for *de novo* prediction. Then, GLEAN (http://sourceforge.net/projects/glean-gene) was used to integrate the results obtained by the two methods. The integrated results were used for subsequent analysis: 1) For functional annotation, diamond software with e value < 1e-5 was used to search genes against Nr (Non-Redundant Protein Sequence Database), KOG (Clusters of Orthologous Groups), Interpro, and SwissProt databases. Interproscan software with parameters--applications Pfam - Gene Ontology (GO) terms was used for GO annotation, and Kobas (KEGG orthology based annotation system) software with parameters default was used for KEGG (Kyoto Encyclopedia of Genes and Genomes) annotation. 2) For repeat components annotation, the assembled genome was compared with the Repbase database using Repetmasker (version 3.3.0; http://www.repeatmasker.org/) to determine the transposon sequence; the tandem repeats were predicted using TRF (http://tandm.bu.edu/trf/trf.html, version 4.04) software, including microsatellites, *etc.*,; 3) For non-coding RNA annotation, tRNAscan-SE software was used for annotation of transfer RNA (tRNA); rRNAmmer software (version 1.2) were used homology prediction and *de novo* prediction of Ribosomal (rRNA); non-coding RNAs such as small nuclear RNA (snRNA) and micro RNA (miRNA) were annotated by the Rfam database (version 14.0; http://rfam.xfam.org/).

### 2.5 CAZYmes annotation of PPL and PCY

Carbohydrate-active enzymes (CAZYmes) in the PPL and PCY genomes were annotated using profile hidden markov models (HMMs, version 2.3.2) by searching against the CAZY database (http://www.cazy.org/). The input data consisted of proteomes of PPL and PCY. A threshold was set for the search results, such that if the alignment length was greater than or equal to 120 amino acids, the E-value had to be less than 1e-5.

### 2.6 Evolution analysis based on nuclear single-copy orthologous genes of PPL and PCY

To analyze the evolution of PPL and PCY among 12 different species, including *P. tuoliensis* (PT), *P*. *eryngii* var. *ferulae* (PC), *P*. *eryngii* var. *eryngii* (PE), *P*. *ostreatus* (PO), *P*. *florida* (PF), *Coprinopsis cinerea* (CC), *Laccaria bicolor* (LB), *Schizophyllum commune* (SC), *Serpula lacrymans* (SL), *Coniophora puteana* (CP). Orthomcl software (version 1.4) was used to cluster gene families and obtain single-copy orthologues (Li et al., 2003). Mafft software (version 7) was used to align all the protein sequences (Katoh and Standley, 2013). RAxML software (random axelerated maximum likelikhood, version 8) was used to construct the phylogenetic relationship of the 12 species using maximum likelihood with 1000 bootstrap runs (Stamatakis, 2014). The mcmctree program in PAML (phylogenetic analysis by maximum likelihood, version 4.4) was used to estimate the divergence time among the 12 species based on the aforementioned phylogenetic tree with three fossil calibrations, CP and SL (70.0–129.4 Mya), LB and CC (59.3–108.4 Mya), and the node formed by the four species (109.9–176.7 Mya) (Jiang et al., 2022).

### 2.7 Whole-genome collinearity analysis of PPL and PCY

Whole genome collinearity analysis of PPL and PCY was performed by the MCScan (JCVI package) (Tang et al., 2008). Firstly, the all-against-all blastp method was used to detect paralogous and orthologous genes from the protein data of PPL and PCY. Then homologous blocks were detected by MCScan with the parameter cscore = 0.99. Finally, the relationship of collinearity between the two genomes was obtained, and subsequent mapping was performed.

### 2.8 Gene family analysis of PPL and PCY

Gene families were identified using an all-against-all blastp method and clustered with the OrthoMCL software (version 1.4) (Li et al., 2003). Expansion or contraction of gene families in each species was investigated using the Cafe software (version 4.2.1) (Bie et al., 2006) with the parameter of -p 0.05. For each gene family, the branch locus model in PAML’s codeml tool (version 4.4; http://abacus.gene.ucl.ac.uk/software/paml.html) was used for positive analysis. KEGG annotation of gene families were conducted by aligning the genes to KEGG database. Blast2GO was used to identify the associated GO terms.

## 3 Results

### 3.1 Genome sequencing and assembly of PPL and PCY

**FIGURE 1:**
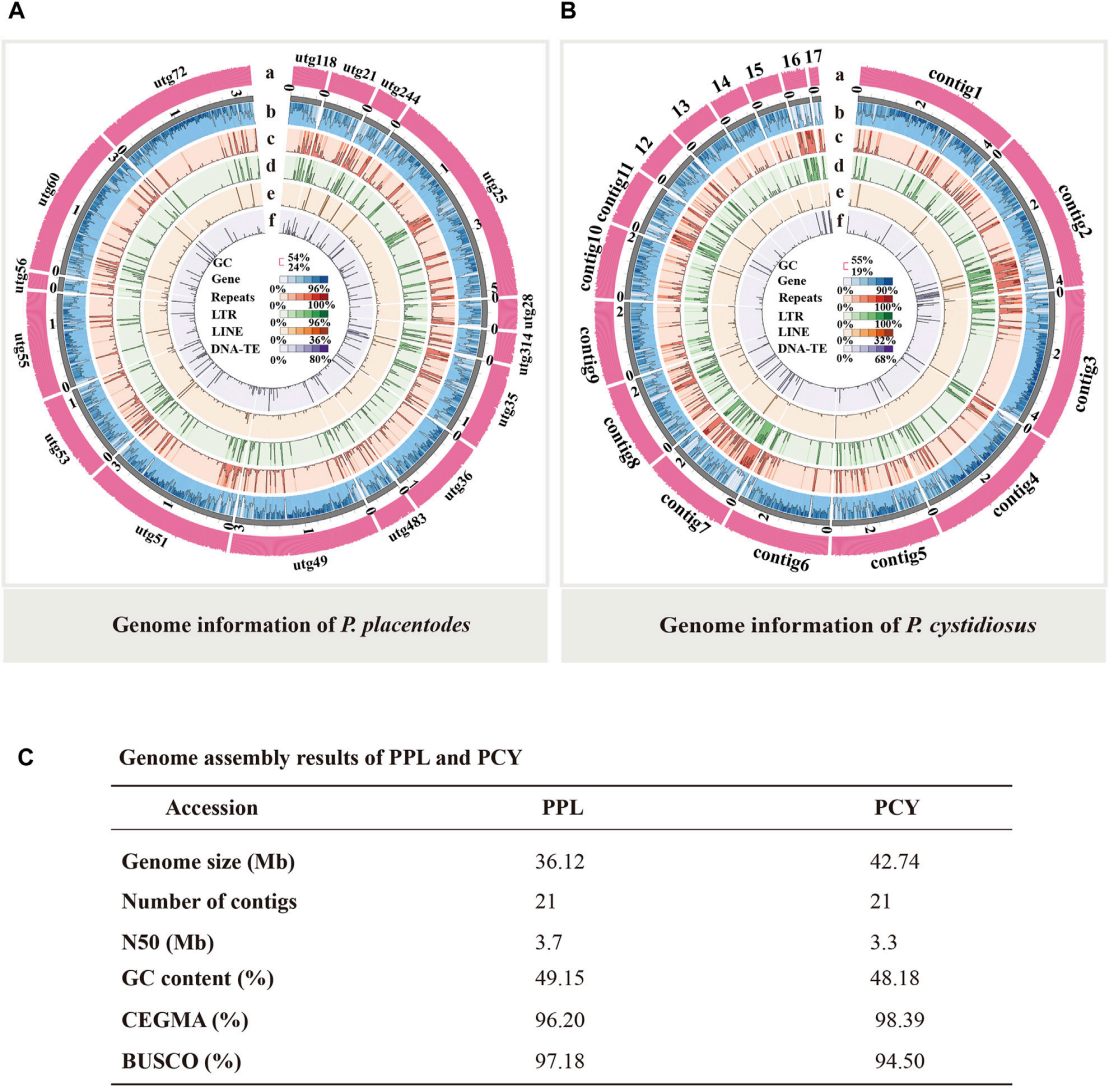
The genome of PPL and PCY. **(A)** The genome information of PPL. **(B)** The genome information of PCY. Outside to inside of concentric circles show assembly contig number and GC content, gene density, all repeat content, LTR content, LINE content, DNA repeat content. **(C)** Genome assembly result of PPL and PCY.

To obtain high-quality genome assemblies for PPL and PCY, we used monokaryon strains obtained through protoplast mononuclearization. We generated 6.7 Gb (186 x) and 4.8 Gb (113 x) of data using the PacBio Sequel platform for PPL and PCY, respectively. The assembly genome size of PPL and PCY was 36.12 and 42.74 Mb, and the contig was all 21. Our assembly also represents good contig N50 length, 3.7 M, and 3.3 M, respectively (Figure 1). CEGMA and BUSCO analysis showed that 96.20% and 98.39% of the core eukaryotic genes and 97.18% and 94.50% single-copy genes were obtained, indicating high quality and integrity for genome assembly of the two species, which provide guarantees to the accuracy of subsequent research.

### 3.2 Genome annotation of PPL and PCY

**FIGURE 2:**
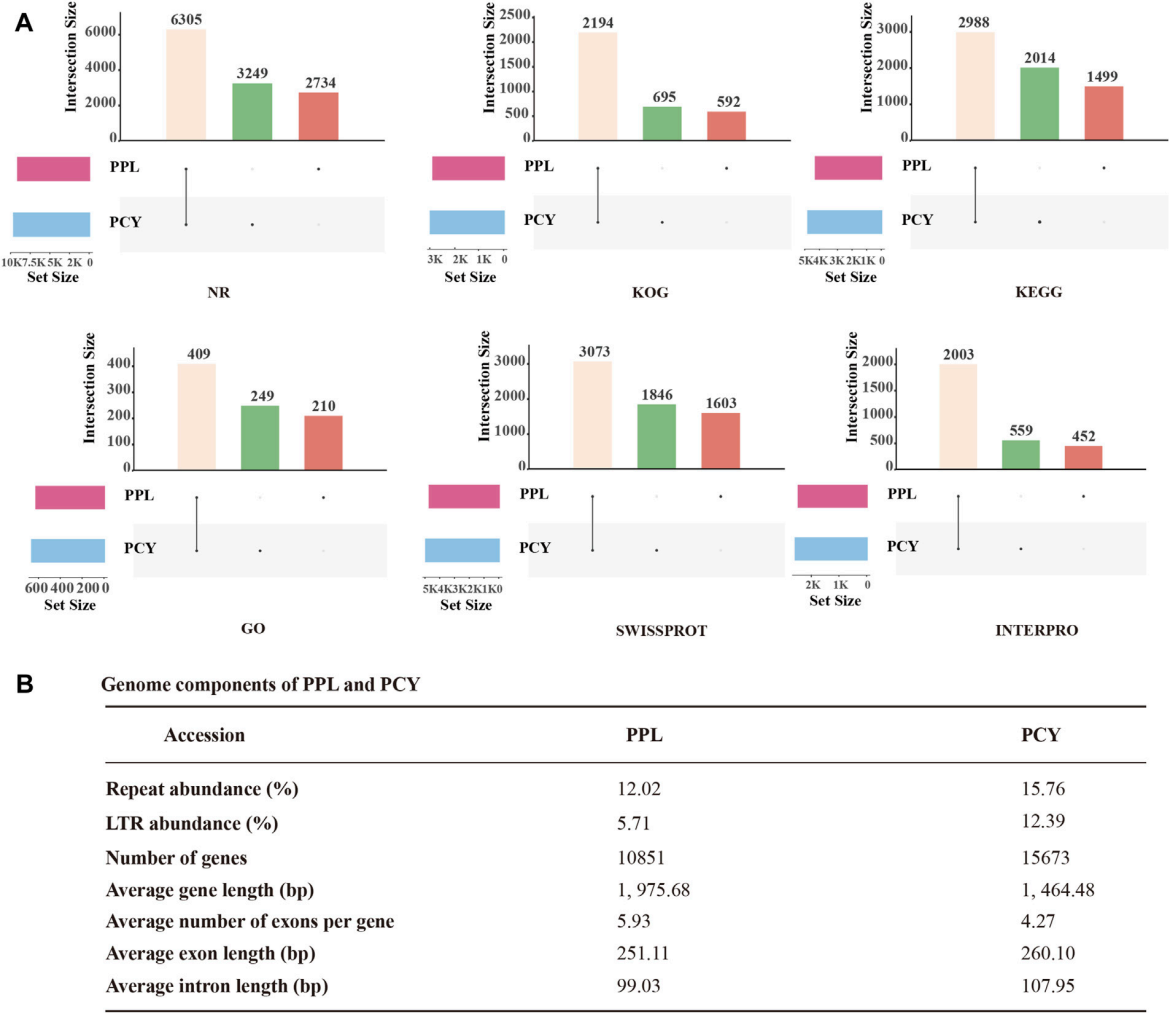
Genome annotation result of PPL and PCY. **(A)** Function annotation of PPL and PCY. Yellow columns represent the same function, green columns represent unique functions in PCY, and brown columns represent unique functions in PPL. **(B)** Genome components of PPL and PCY.

To more accurately predict PPL and PCY protein-coding genes, we performed homologous annotation and *denovo* annotation. The result showed that PPL and PCY genomes include 10,851 and 15,673 protein-coding genes, accounting for 59.34% and 53.70% of the genome size, respectively (Figure 2B). The predicted average length of protein-coding genes was 1, 975.68 bp and 1, 464.48 bp, 5.93 and 4.27 exons per gene on average, respectively. The average exons length was 251.11 and 260.10 bp, and the average intron length was 99.03 and 107.95 bp. We further annotated these genes by Nr, InterPro, GO, KEGG, Swiss-prot, KOG databases. Among them, 10,445 (96.26%), 6,027 (55.54%), 2,544 (23.44%), 5,652 (52.09%), 5,862 (54.02%), 4,025 (37.09%) were annotated in PPL and 11,617 (74.12%), 6,625 (42.27%), 2,849 (18.18%), 6,564 (41.88%), 6,286 (40.11%), 4,237 (27.03%) were annotated in PCY (Figure 2A, Supplementary Table S1). It is noticed that the gene terms annotated by PPL in six functional databases were all significantly less than PCY, indicating PPL genome experienced significant gene contraction during evolution. In addition, both PPL and PCY contain their own unique functional genes, accounting for 18%–34% and 22%–62% of the annotated genes in different databases, respectively. Particularly, the percentage of unique genes annotated in GO and KEGG databases is the highest, indicating PPL and PCY have undergone significant gene differentiation during the evolutionary process.

We identified ∼4.3 M and 6.7 M repeat sequences in PPL and PCY, which accounted for 12.02% and 15.76% of their respective genome sizes (Figure 2B). Among them, tandem repeats (TRF) sequence length is 0.41 M (1.14%) and 0.37 M (0.86%), transposable element (TEs) sequence length was 4.1 M (11.26%) and 6.4 M (14.89%). Among the different TE types, long terminal repeats (LTRs) were most abundant (2.1 and 5.3 Mb), accounting for 5.71% and 12.39% of the genome size, followed by DNA transposons (0.4 and 0.6 Mb), long interspersed nuclear elements (LINEs) (0.24% and 0.16%), and short interspersed elements (SINEs) (0.004% and 0.02%) (Supplementary Table S2).

We also identified non-coding RNA in PPL and PCY. A total of 173 tRNAs and 58 rRNAs were identified in PPL, while 165 tRNAs and 102 rRNAs were identified in PCY. In addition, 7 and 12 tRNA pseudogenes were predicted in PPL and PCY, respectively. We also predicted 56 miRNA and 14 snRNAs containing 9 splicing RNAs and 5 C/D nucleolar small RNAs in PPL, and 17 miRNA and 14 snRNAs containing 10 splicing RNAs and 4 C/D nucleolar small RNAs in PCY (Supplementary Table S3).

### 3.3 CAZYmes analysis of PPL and PCY adapted to different substrate

**FIGURE 3:**
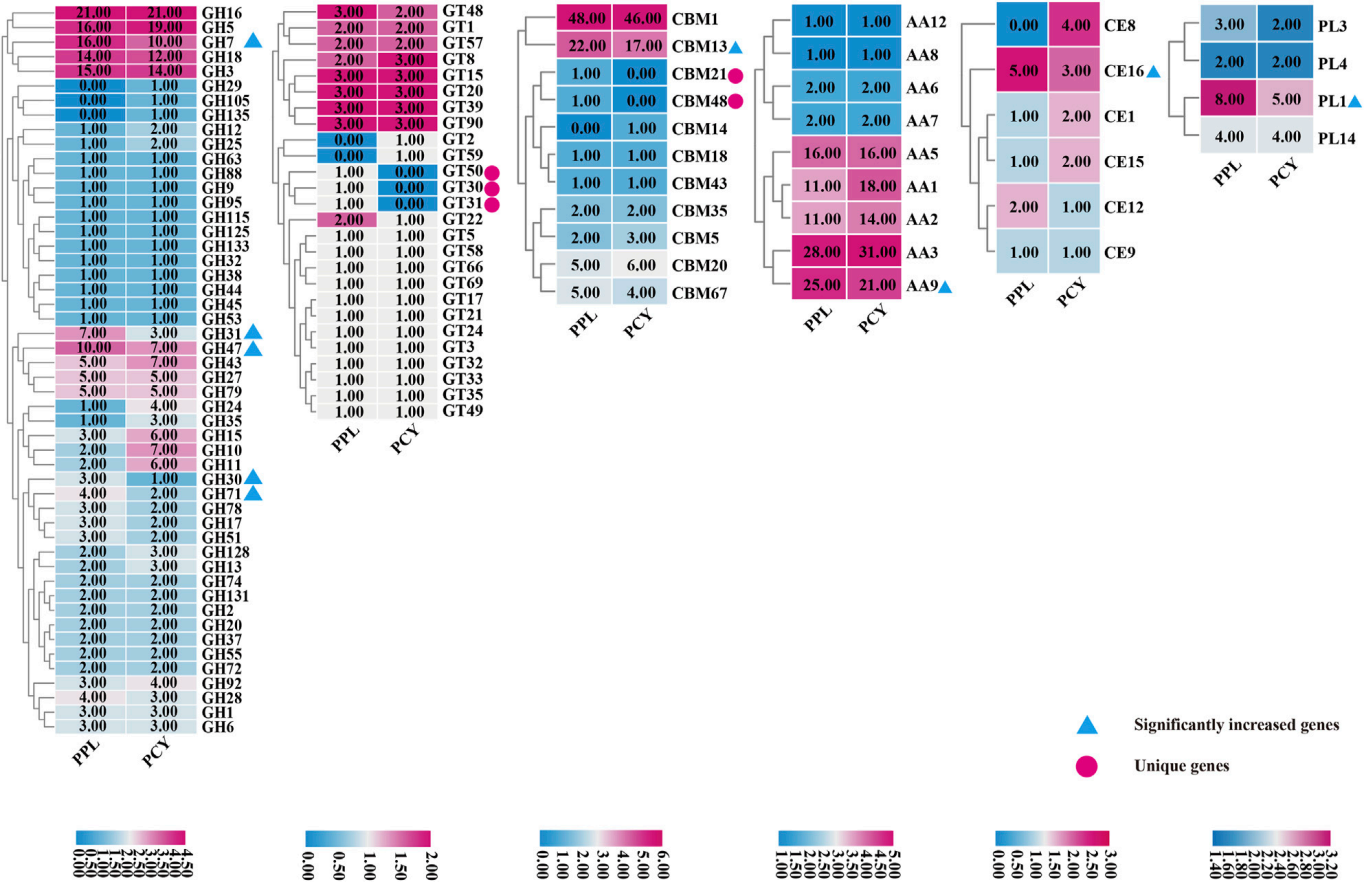
Comparison of CAZYmes annotated by PPL and PCY. From left to right, show GHs, GTs, CBMs, AAs, CEs, and PLs. White numbers represent the number of annotated genes. The chromaticity bar at the bottom represents the gene number of different colors, corresponding to the figure above.

To study the difference in composition of enzymes mainly related to lignocellulose degradation, we conduct comparative CAZYmes analysis of PPL and PCY. A total of 434 and 439 CAZYmes genes were annotated in PPL and PCY genome using hmmer software, respectively (Figure 3). Glycoside Hydrolases (GHs) was the most annotated CAZYmes gene family in these two *Pleurotus* species, accound for 184 (42.4%) and 190 (43.3%) of these two species, followed with the Auxiliary Activities (AAs) and Carbohydrate-Binding Modules (CBMs), annotated to 97 (22.4%) and 88 (20.3%) in the PPL, and 106 (24.1%) and 81 (18.5%) in PCY. The number of genes annotated in GlycosylTransferases (GTs), Carbohydrate Esterases (CEs) and Polysaccharide Lyases (PLs) was relatively small, annotated to 38 (8.8%), 10 (2.3%) and 17 (3.9%) in PPL and 36 (8.2%), 13 (3.0%) and 13 (3.0%) in PCY. Further comparison with the PCY found that, AA9, CBM13, CE16, GH30, GH31, GH47, GH7, GH71, and PL1 were significantly increased, and CBM21, CBM48, GT30, GT31, and GT50 were unique CAZYmes genes in PPL. PCY contains more AA1, GH10, GH11, GH15 CAZYmes and CBM14, CE8, GH105, GH135, and GT2 were unique CAZYmes gene family. We identified differences in the number of specific pectin lyase gene modules and genes in the genomes of PPL and PCY. Both fungi have four gene modules for pectin lyase, including PL1, PL3, PL4, and PL14. While PL4 and PL14 contain the same number of genes in both fungi, PPL has a greater number of genes for PL1 and PL3 compared to PCY. Specifically, PPL has a total of 8 copies of the PL1 gene and 3 copies of the PL3 gene, while PCY has 5 copies of the PL1 gene and 2 copies of the PL3 gene.

#### 3.3.1 Cellulose and hemicellulose

Although the main components of hemicellulose are xylan and mannan, the composition of hemicellulose in coniferous wood is quite different from that in broad-leaved wood. Firstly, coniferous tree xylan do not contain acetyl groups, but have arabinose branched chains that require corresponding debranching enzymes for degradation. A total of 91 GHs (PPL 45 and PCY 46) with related functions were annotated in PPL and PCY, namely, GH1, GH5, GH30, GH43, and GH51. Among them, no difference occured in the number of GH1 enzymes. GH3, GH30, and GH51 in PPL contain α-L-arabinofuranosidase (EC 3.2.1.55) and glucuronoarabinoxylan endo-β-1,4-xylanase activities, while GH5 and GH43 in PCY contain L-arabiofuranosidase activities, which indicating PCY also has the ability to degrade hemicellulose of coniferous wood, and hemicellulose degrading enzyme is not the main reason for determining whether fungi can grow on coniferous substrate. Secondly, in general, in addition to glucose and mannose, mannan in coniferous trees also contains acetyl- and galactose branched chains, which are absense in broad-leaved trees. Compared to PCY, GH31 (α- The number of N-acetylgalactosaminidase (EC 3.2.1.217) in PPL is 2.5 times than PCY. In addition, CBM1, CBM5, CBM13, CBM35, and CBM43 all have the ability to bind and assist in the degradation of cellulose or hemicellulose. PPL contains 75 genes, and PCY contains 69 genes, indicating PPL has more advantages in the co-enzyme of cellulose degradation. The number of GTs is relatively large in PPL, especially three unique enzymes related to mannose transfer, GT30, GT31, and GT50, which assist in the degradation of mannan. No quality difference in AA7 and AA8 related to cellulose degradation, while AA1, AA2, AA3, and AA9 with differences are Laccase, Manganese peroxide, cellobiose dehydrogenase, and lytic polysaccharide monooxygenases, all of which are related to lignin degradation. In conclusion, although the number of cellulose and hemicellulose degradation related enzymes in PCY and PPL is similar, the number of co-enzymes in PPL is significantly more than that in PCY. Therefore, PPL has the ability to degrade cellulose and hemicellulose faster.

#### 3.3.2 Pectin

Plant pectin is mainly composed of Homogalactiuronan and Rhamnogalactiuronan. A total of 50 enzymes (PPL 27 and PCY 23) related to the degradation of these two substances were annotated, namely, GH28, GH78, CBM67, CE8, CE12, PL1, PL3, and PL4, which indicating PPL has a higher pectin degradation ability than PPL. Among the 50 enzymes, the number of PL4 genes is consistent in PPL and PCY, CE8 is a unique pectin methylesterase in PCY, and the remaining enzymes are more abundant in PPL than in PCY. Homogalacturonan is a polymer which was composed of galacturonic acid residues connected by α- 1,4-linked. GH28 is endopolygalacturonases, which can cleave the α-1,4-linkage. PL1 and PL3 are two pectate lyase, which can degrade the α-1,4-linkage between galacturonic acid residues at the non-reducing end of homogalacturonan. In terms of rhamnogalacturonan degradation, the main enzymes involved include GH78, CBM67, CE8, and CE12. Among them, GH78 isα-L-rhamnosidase enzymes, which can act on the terminal non reducing end of the rhamnogalacturonan backbone. CBM 67 can bind L-rhamnose and enhance the degradation rate of rhamnose. CE12 and CE8 are pectin acetyl esterases and pectin methylesterase, which target the backbone of rand homogalacturonan, respectively.

#### 3.3.3 Lignin

Lignin is an important component of plant cell walls, which content is higher in coniferous trees than broad-leaved trees. Therefore, laccase, manganese peroxidase and lignin peroxidase, which are mainly related to lignin degradation, have also been annotated. A total of 70 enzymes related to lignin degradation were annotated, including 40 in PPL and 30 in PCY. The quantity in PCY is much higher than that in PPL, indicating PCY has stronger lignin degradation ability, and also reflected enzymes related to lignin degradation are not the main reason why PPL adapts to coniferous tree substrates.

### 3.4 Molecular evolution of PPL and PCY

To analysis the evolution and differentiation of PPL and PCY, we performed phylogenetic analysis based on nuclear single-copy orthologous genes of PPL, PCY and 10 other reported strains, contains *P. tuoliensis* (PT), *Pleurotus eryngii* var. *ferulae* (PC), *P*. *eryngii* var. *eryngii* (PE), *P*. *ostreatus* (PO), *P*. *florida* (PF), *Coprinopsis cinerea* (CC), *Laccaria bicolor* (LB), *Schizophyllum commune* (SC), *Serpula lacrymans* (SL), *Coniophora puteana* (CP) (Table 1)*.* We used maximum likelihood method to construct a phylogenetic tree with CP and SL as outgroup. We obtained 1,869 nuclear single-copy orthologous genes and used them to construct the phylogenetic tree after sequence aligment. Based on the phylogenetic tree, PCY adapted to broad-leaved tree substrate was the earlist to differentiate with the divergence time 111 million years ago (MYA), followed by PPL adapted to conifers substrate with the divergence time 62.7 MYA (Figure 5A). Other species PE, PC, PT adapted to Apiaceae plants and PO, PF adapted to broad-leaved substrate with the divergence time less than 29.9 MYA. From 111 to 66.7 million years, the period was in the Cretaceous period, during which the temperature rises and the vegetation was luxuriant, which was suitable for the high growth temperature type of PCY. Therefore, we speculate the climatic conditions promote the differentiation of PCY. Differently, PPL was mostly collected from subalpine areas, and its differentiation may be related to orogeny in the late Cretaceous. In addition, gymnosperms originated in the late Devonian period, at least 350 million years ago. There was no significant correlation between the occurrence time of known *Pleurotus* mushrooms and conifers or broad-leaved trees in history.

**TABLE 1:** List of genomes used in current study.

Abbreviation	Scientific name	Sequence type	Resource
PPL	*P. placentodes*	Protein	This study
PCY	*P. cystidiosus*	Protein	This study
PT	*P. tuoliensis*	Protein	NCBI
PC	*P. eryngii var. ferulae*	Protein	Dai et al. (2019)
PE	*P. eryngii var. eryngii*	Protein	MushDB
PO	*P. ostreatus*	Protein	MushDB
PF	*P. florida*	Protein	MushDB
CC	*C. cinerea*	Protein	NCBI
LB	*L. bicolor*	Protein	NCBI
CP	*C. puteana*	Protein	NCBI
SL	*S. lacryman*	Protein	NCBI
SC	*S. commune*	Protein	NCBI

### 3.5 Whole-genome collinearity analysis of PPL and PCY

**FIGURE 4:**
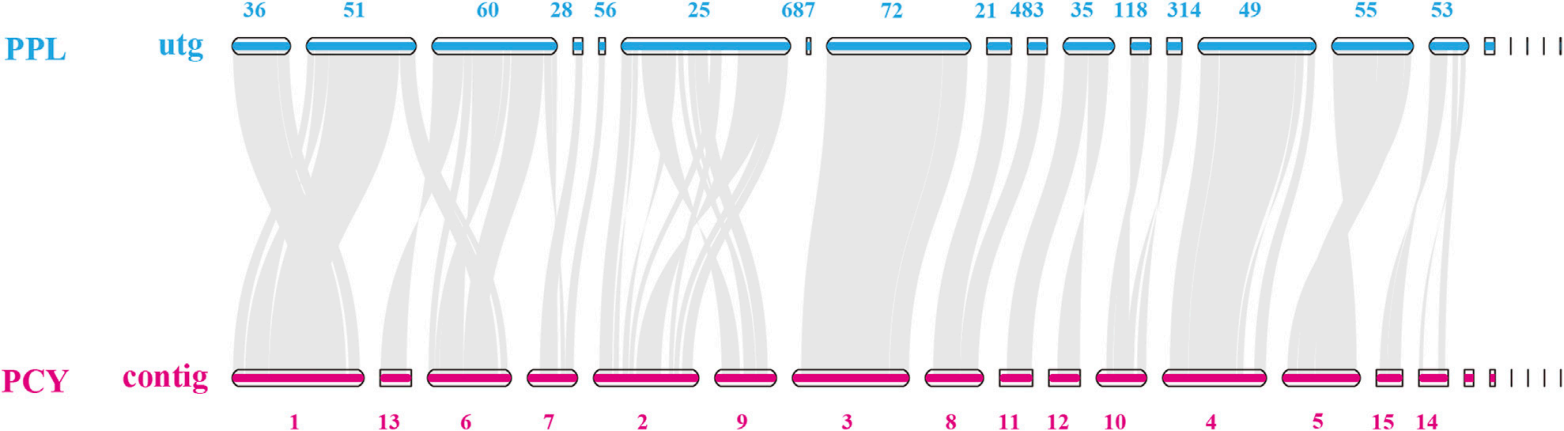
Genome collinearity analysis of PPL and PCY. Note: The contig/utg number is not continuous due to subsequent assembly optimization.

To investigate the degradation ability of different substrates of the *Pleurotus* from the genomic level, we conducted a collinearity analysis of the genomes of the PCY adapted to the broad-leaved tree and the PPL adapted to the coniferous tree (Figure 4). The results showed that the alignment rate of homologous fragments between each contig of PPL and PCY strains was relatively high, in which the contig36, contig28, contig56, contig72, contig214, contig83, contig49, and contig55 were basically consistent with the gene sequence arrangement on their corresponding contigs in PCY. And a number of contigs showing inversions, such as contig51 in PPL and contig1 in PCY, contig 60 in PPL and contig6 in PCY, contig 25 in PPL and contig2, contig9 in PCY. In addition, five shorter, one-gene-only and unmatched contig both in PPL and PCY.

Further sequence analysis revealed mitochondrial genome sequences in utg18 and contig76 of PPL and PCY, respectively. Among the remaining contigs, several in the PPL genome contained putative flavin, cytochrome C oxidase subunit 2, and cytochrome P450 genes, but no genes were annotated in PCY. These segments had a relatively high GC content and high repetitive sequences.

We also annotated 1,055 genes in contig1 of PCY and 1,648 genes in utg51 of PPL (Supplementary Table S4), and found that the types of metabolic pathways were similar in both species. However, there were significant differences in the number of genes in some pathways of common metabolic pathway. PPL had a large number of stress-related genes in pathways related to chemokine signaling, geraniol degradation, ABC transporters, degradation of aromatic compounds, fatty acid metabolism, naphthalene degradation, and starch and sucrose metabolism. We also found significant expansion of 1,337 genes in utg25 and 1,756 genes in contig2 and contig9 of PPL and PCY, respectively (Supplementary Table S5).

### 3.6 Comparative genomic analysis of PPL and PCY

#### 3.6.1 Gene family cluster

**FIGURE 5:**
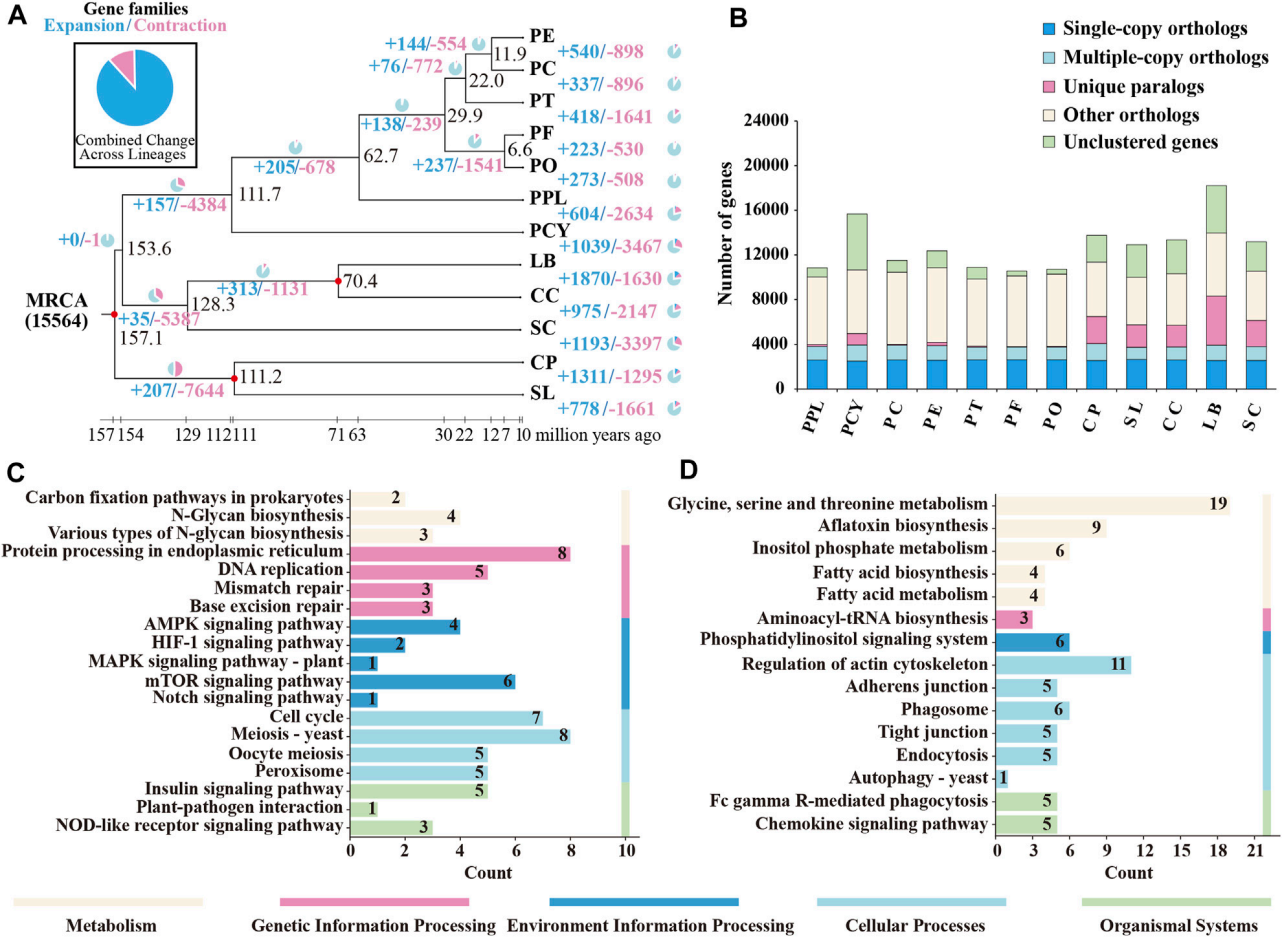
Comparative genome analysis of PPL and PCY. **(A)** Expansion and contraction of gene families in 12 species genomes. The numbers of gene families that expanded (blue) or contracted (red) in each lineage after classification are down on the corresponding branch. MRCA, the most recent common ancestor. The time shown in the bars represents the estimated divergence time. **(B)** Analysis of common unique gene families. The different color represent different orthologs. **(C)** KEGG enrichment of positively selected genes in PPL. **(D)** KEGG enrichment of expansion genes in PPL. The bottom color bar represents different KEGG pathway classifications.

We selected PPL, PCY, and 10 other species for gene family clustering, which revealed that the 12 species shared 3,052 gene families, with PPL and PCY having 61 and 306 unique gene families, respectively (Figure 5B). The PPL-specific gene families mainly related to immune and signaling pathways, such as PI3K-Akt signaling pathway, chemokine signaling pathway, and MAPK signaling pathway. The PCY-specific gene families mainly related to DNA replication, pyrimidine metabolism, purine metabolism, sesquiterpenoid and triterpenoid biosynthesis. We also identified gene families associated with pigment synthesis also have been identified, such as tyrosine and tryptophan metabolism, and glutathione metabolism.

#### 3.6.2 Expansion and contraction of gene family

To identify the evolutionary dynamics of the gene family, we used the CAFE software to analyze gene family expansion, and a *p*-value < 0.05 was considered to be significantly expanded or contracted. We found that 604 gene families had evolved to expand in PPL, with 18 significantly expanded gene families. Among the PCY, 1039 gene families had evolved to expand, with 16 significantly expanded gene families (Figure 5A). KEGG and GO analysis showed that the PPL expansion gene family was significantly enriched in metabolic pathways such as fatty acid biosynthesis, chemokine signaling pathway, Fc gamma R-mediated phagocytosis, aminoacyl-tRNA biosynthesis, glycine, serine and threonine metabolism, while the function of these family genes was closely related to the stress resistance process (Figure 5D). Lysine degradation and phosphatidylinositol signaling system metabolic pathway related genes were significantly enriched in the expanded gene family of PCY. Furthermore, we identified 2,634 contracted gene families in PPL, of which only 3 were found to be significantly contracted. Similarly, we found 3,467 contracted gene families in PCY, but only 1 was significantly contracted.

#### 3.6.3 Positively selected genes identification

We used the branch-site model calculation and likelihood ratio test (*p* < 0.05) to identify 264 and 496 positively selected genes in PPL and PCY, respectively. GO and KEGG analysis showed that the positively selected genes were significantly enriched in peroxisome, signaling pathway, AMPK signaling pathway, MAPK signaling pathway, protein processing in endoplasmic reticulum and other metabolic pathways in PPL (Figure 5C). On the other hand, the genes involved in PCY were significantly enriched in riboflavin metabolism, fructose and mannose metabolism, glycolysis/gluconeogenesis and other metabolic pathways. Furthermore, we identified metabolic pathways related to antibiotic synthesis, such as neomycin, kanamycin and gentamicin biosynthesis, carbapenem biosynthesis, streptomycin biosynthesis, in the PCY genome.

## 4 Discussion

*Pleurotus* species are widely distributed throughout the world and are highly valued for their economic and culinary uses. While 18 strains of *Pleurotus* genomes have been published in NCBI and MushDB databases (Fu et al., 2022), the genomes of PPL and PCY have not been reported until this study. PPL is a rare species of *Pleurotus* that was collected from Picea, a coniferous substrate found in Subalpine Forests. This suggests that PPL has the potential to thrive on similar substrates. Given the growing issue of environmental damage caused by large amounts of conifer waste, the use of PPL as an edible mushroom resource could be particularly valuable. PPL’s ability to utilize coniferous substrates effectively could help address this issue by providing a sustainable means of utilizing such waste. Therefore, the discovery of PPL’s potential as an edible mushroom resource has practical applications beyond its scientific significance. In this research, two high-quality reference genomes for *Pleurotus* were assembled and annotated with genome sizes of 36.12 and 42.74 Mb, adapted to broad-leaved tree and conifer substrates, respectively. This study has enriched the genetic information of *Pleurotus* species, providing insight into the evolution and conifer utilization.

The phylogenetic relationship of *P. ostreatus* species complex has always been a controversial issue due to its phenotype being greatly influenced by the environment. Compared to the traditional use of ITS, RPB2 and other fragments, phylogenetic analysis of *Pleurotus* was conducted from a genomic perspective in this study, which has the advantages of more comprehensive and accurate information. Our phylogenetic analysis revealed that PPL is more closely related to *P*. *ostreatus*, while PCY is relatively distantly related to other *Pleurotus* species. This is consistent with the results obtained using the ITS bar code by Liu et al. (2016). Furthermore, we predicted the differentiation time of PCY and PPL, and found that the differentiation time of these two species was 111 and 62.7 MYA, respectively. Species differentiation are general influenced by climate conditions, geographical isolation, and substrate. However, fossil records indicate the existence of the Pinaceae in the Early Jurassic, and the Betulaceae, Fagaceae, and Salicaceae in the Middle Cretaceous (Lin et al., 2010; Li et al., 2020), while the PPL differentiated from the Cretaceous and grew in mixed broadleaf-conifer forest. Additionally, a monoterpene found in coniferous plants with bactericidal effects (Li et al., 2017), suggests the potential ability of PPL and PCY to degrade coniferous substrates. ABC transporter also has been identified as a key element against host defenses in other fungi, such as *Phlebiopsis gigantea* (Hori et al., 2014). Moreover, PPL’s ability to grow in subalpine areas with high altitudes suggests the involvement of fatty acid metabolism, which has been previously linked to plateau adaptation in the Himalayan marmot genome (Bai et al., 2019). It is worth noting that PPL mainly collected from subalpine regions, and 62.7 MYA was in the late Cretaceous orogenic period. These findings suggest that climate conditions and geographical isolation maybe the main factors to induced its differentiation. Our study also identified PPL and PCY as two valuable resources for exploring the genetic mechanisms underlying substrate adaptation in *Pleurotus* mushrooms. We found that both species have undergone significant gene family expansions, with PPL showing a greater degree of expansion on coniferous substrates and PCY exhibiting more expansion on broadleaf substrates. Additionally, our whole-genome collinearity analysis suggests that adaptive evolution has occurred at the genome level in both PPL and PCY.

The plant cell wall is mainly composed of lignin, cellulose, hemicellulose and pectin, which require specific enzymes (CAZYmes) for degradation. Coniferous substrates have more lignin than broad-leaved tree substrates, which makes the role of glycoside hydrolase in lignocellulose degradation limited (Zhang et al., 2006). However, the synergy of lytic polysaccharide monooxygenase (LPMO) and glycoside hydrolase (GHs) can improve lignocellulose degradation (Harris et al., 2010; Li et al., 2022). The study identified a higher number of genes related to lignocellulose degradation in conifers, such as AA9 (LPMO), GH30 (glucuronoarabinoxylan endo-β-1,4-xylanase) and GH31 (β-glucosidase), GH47 (α-mannosidase), GH7 (cellobiohydrolase), and GH71 (glucanase), which may be attributed to the high lignin content present in these trees. However, the number of enzyme genes related to lignin degradation in PPL is significantly less than that in PCY, indicating lignin degradation is not the main reason for PPL’s adaptation to coniferous substrate. It is worth notingg that the number of cellulose and hemicellulose degradation related enzymes in PCY and PPL is similar, but PPL contains more co-enzymes, indicating PPL has the ability to degrade cellulose and hemicellulose faster. Similarly, the degradation of coniferous substrates is also been reported facilitated by the crucial roles played by β-glucosidase and cellobiohydrolase in their early decomposition, as established by studies on penicillium and endophytic fungi (Yuan and Chen, 2014; Rai et al., 2016).

Pectin is one of the main components to make up plant cells, which was composed of homogalactiuronan and rhamnogalactiuronan. In cell wall degradation, pectin degradation is earlier than cellulose and hemicellulose, and cellulose and hemicellulose directional hydrolase genes with the highest transcriptional abundance level come from GH1, GH3, GH5, GH7, GH12 and GH30 CAZyme families (Blackman et al., 2015). In this study, the high levels of pectin degrading enzymes in PPL, such as PL1, PL3, CE12, may facilitate the degradation of coniferous substrates (Atanasova et al., 2018), which could promote effective colonization. In addition, we also noted that CE8 is missing in PPL. CE8 is a kind of pectin methylesterases, mediating the removal of methyl esters from homogalacturonan process and releases methanol, which can be used as a signal molecule to cause the response of plants stress resistance, further changing the activity of pectin degrading enzymes such as polygalacturonase. The lack of CE8 in PPL may reduce plant stress resistance and increase survivability (Yang et al., 2013). To sum up, the number of pectinase and cellulase annotated from a genomic perspective was significantly higher in PPL than PCY, confirming our hypothesis that one of the factor for PPL adapts to coniferous tree maybe by rapidly plant cell walls degradation.

Conifers are rich in galactoglucomannan, a complex polysaccharide composed of mannosyl (Terrett et al., 2019) and glucosyl residues (Hannuksela and Hervé du Penhoat, 2004). PPL expresses specific genes, namely, GT50 (mannosyltransferase) and CE16 (acetylesterase), which are essential in breaking down coniferous galactoglucomannan. The unique enzymes GT30, GT31, and GT50 in PPL that are associated with mannose transfer all assist in the degradation of mannan. In addition, the GT family is closely associated with microbial extracellular polysaccharide synthesis (Wang et al., 2003; Rollefson et al., 2011; Xu et al., 2021b), which may play a crucial role in colonizing and interacting with hosts (Chandrasekar et al., 2022). With more annotated GT genes and specific glycosyltransferases, PPL has a stronger extracellular polysaccharide synthesis capacity, making it potentially adept at colonizing conifer substrates. The study also identified auxiliary enzymes, such as CBM13 and CBM21, which can enhance the cellulase degradation ability.

The ability of PPL to tolerate coniferous substrate can be attributed to its immune anti-fungal effect, as demonstrated in previous research (Chen et al., 2002). When comparing PPL to PCY, it was found that PPL had significant expansions in immunity and signal pathways, such as geraniol degradation and fatty acid biosynthesis, through gene family analysis. Geraniol, a terpenoid found in coniferous plants, has been shown to have strong bactericidal effects on various pathogens, including *Drechslera oryzae*, at concentrations as low as 0.2 μL/mL (Li et al., 2017; Kaur et al., 2019; Scariot et al., 2021). The expansion of geraniol degradation metabolism in PPL may be one of the main factors contributing to its adaptability to conifer substrates.

Furthermore, the tolerance of fungi is largely achieved by controlling growth and development and maintaining cell membrane stability (Gu and Wei, 2010; Xie et al., 2011). In this study, MAPK signal pathway, AMPK signal pathway, peroxisome, and fatty acid biosynthesis were identified and were proven to regulate cellulase genes (Zhang, 2015), plant-pathogen interactions (Kubo, 2013), pesticide tolerance (Chen, 2001), pathogenicity (Wang et al., 2022), morphogenesis (Yao et al., 2019), and environmental adaptability (Tasseva et al., 2004; Fu et al., 2016), potentially being closely related to this function. Other stress resistance-related metabolisms were also identified, including chemokine signaling pathway, ABC transporters, Fc gamma R-mediated stress, aminoacyl tRNA biosynthesis, glycine, serine, and threonine metabolism. These have been shown to be related to microbial infection (Chensue, 2001), monoterpene resistance (Wang et al., 2013; Kligun et al., 2017), and exogenous oxidative stress (Qi et al., 2019).

Compared to PCY, PPL has fewer metabolic pathways related to terpene, pigment, and antibiotic synthesis. However, some important terpenoid biosynthesis pathways, such as diterpenoid biosynthesis and sesquiterpenoid and triterpenoid biosynthesis, are present in PPL. Terpenes are primarily synthesized through sesquiterpenoid and triterpenoid biosynthesis, with isopentenyl diphosphate (IPP) serving as a common precursor that can form various terpenoid precursors via isopentenyl transferase. Terpene synthase then catalyzes the formation of different terpenoid skeletons, such as monoterpenes, sesquiterpenes, diterpenes, dipsesquiterpenes, and triterpenes (Wang et al., 2019b). The annotated pathways related to terpenoid biosynthesis in PPL provide a genetic foundation for the synthesis of terpenoids in this species at the genomic level.

The metabolism of fungal pigments, such as L-dopa and DHA, is an important area of research, and tyrosine metabolism has been identified as closely related to the formation of fungal melanin. Tyrosinase is a crucial enzyme in the L-dopa pathway of *Agaricus bisporus*, and tyrosine forms DOPA under the action of tyrosinase. It then reacts with polyphenoloxidase and other enzymes to form melanin (Weijn et al., 2013). Although there are fewer tyrosine metabolism-related genes in PPL compared to PCY, it is possible that the dark color of PPL’s fruiting body is related to the metabolism of tyrosine.

In addition to the selection of metabolic pathways related to growth and development, PPL has fewer metabolic pathways related to antibiotic synthesis than PCY. However, PPL still has the potential to produce natural antibiotics, such as cephalosporin biosynthesis and beta-lactam biosynthesis. These pathways indicate that PPL has medicinal value and may have evolved adaptive strategies to ensure survival in its specific environment. Overall, PPL and PCY have evolved adaptively to different substrates, and genes related to stress resistance, substrate utilization, growth, and development have been positively selected to ensure their survival in diverse environments. However, it is undeniable that the current work has only conducted mining and analysis at the genomic level, and we will further conduct cultivation and expression levels validation in subsequent work.

## 5 Conclusion

Exploring the adaptability of species to coniferous substrate is an important prerequisite for the development and utilization of coniferous waste resources. In this study, we provided two genomes of *Pleurotus* that adapt to coniferous (PPL) and broad-leaved substrate (PCY), and carried out phylogenetic and coniferous adaptability studies respectively. Species evolution studies reflect PPL and PCY have undergone significant differentiation in the genome. PPL is closer to the traditional *Pleurotus* species and differentiates from 62.7 MYA, which is 49 MYA later than PCY (111.7 MYA). Comparative CAZYmes between PPL and PCY indicate PPL has an advantage in the quantity of enzymes related to cellulose and pectin degradation, despite coniferous substrate contains higher lignin content than broad-leaved substrate, which may providing favorable advantage for faster coniferous substrate colonization. In addition, comparative genomic analysis identified geraniol degradation and peroxisome pathway should play an important role for PPL’s tolerance to conifer substrates in stress resistance. These findings provide valuable genetic resources for understanding the evolution and coniferous adaptation of *Pleurotus* species from the genomic level, and lay the foundation for developing new varieties of coniferous substrate utilization.

## Data Availability

All the genome sequences have been submitted to Figshare database: https://figshare.com/projects/Genome_assembly_and_annotation_of_two_Pleurotus/184330.

## References

[B1] Araújo N. L. Avelino K. V. Halabura M. I. W. Marim R. A. Kassem A. S. S. Linde G. A. (2021). Use of green light to improve the production of lignocellulose-decay enzymes by *Pleurotus* spp. in liquid cultivation. Enzyme Microb. Technol. 149, 109860. 10.1016/j.enzmictec.2021.109860 34311876

[B2] Atanasova L. Dubey M. Grujić M. Gudmundsson M. Lorenz C. Sandgren M. (2018). Evolution and functional characterization of pectate lyase PEL12, a member of a highly expanded *Clonostachys rosea* polysaccharide lyase 1 family. BMC Microbiol. 18, 178. 10.1186/s12866-018-1310-9 30404596 PMC6223089

[B3] Bai L. Liu B. Ji C. Zhao S. Liu S. Wang R. (2019). Hypoxic and cold adaptation insights from the Himalayan marmot genome. IScience 11, 519–530. 10.1016/j.isci.2018.11.034 30581096 PMC6354217

[B4] Berkeley M. J. (1852). Decades of fungi, XXXVII, XXXVIII. Sikkim and Khassya fungi. Hooker’s J. Bot. Kew Gard. Misc. 4, 97–107.

[B5] Bie T. D. Cristianini N. Demuth J. P. Hahn M. W. (2006). CAFE: a computational tool for the study of gene family evolution. Bioinformatics 22, 1269–1271. 10.1093/bioinformatics/btl097 16543274

[B6] Blackman L. M. Cullerne D. P. Torreña P. Taylor J. Hardham A. R. (2015). RNA-seq analysis of the expression of genes encoding cell wall degrading enzymes during infection of lupin (*Lupinus angustifolius*) by *Phytophthora parasitica* . PLoS One 10, e0136899. 10.1371/journal.pone.0136899 26332397 PMC4558045

[B7] Chandrasekar B. Wanke A. Wawra S. Saake P. Mahdi L. Charura N. (2022). Fungi hijack a ubiquitous plant apoplastic endoglucanase to release a ROS scavenging β-glucan decasaccharide to subvert immune responses. Plant Cell. 34, 2765–2784. 10.1093/plcell/koac114 35441693 PMC9252488

[B8] Chen S. Y. Wu Q. P. Zhou X. Y. Que S. H. Qiu W. Y. (2002). Research progress on cultivation of edible fungi with conifer sawdust. Microbiol. China 02, 49–52. 10.13344/j.microbiol.china.2002.02.014

[B9] Chen S. W. (2001). Correlation of catalase and peroxidase with pesticide tolerance in massonpine caterpillar. Acta Entomol. Sin. 44, 9–14. 10.16380/j.kcxb.2001.01.002

[B10] Chensue S. W. (2001). Molecular machinations: chemokine signals in host-pathogen interactions. Clin. Microbiol. Rev. 14, 821–835. 10.1128/CMR.14.4.821-835.2001 11585787 PMC89005

[B11] Croan S. C. (2004). Conversion of conifer wastes into edible and medicinal mushrooms. For. Prod. J. 54, 68–76.

[B12] Dai Y. T. Sun L. Yin X. L. Gao M. Zhao Y. Jia P. (2019). *Pleurotus eryngii* genomes reveal evolution and adaptation to the Gobi desert environment. Front. Microbiol. 10, 2024. 10.3389/fmicb.2019.02024 31551962 PMC6734163

[B13] Fu Y. P. Liu Y. Dai Y. T. Yang C. T. Duan M. Z. Zhang Z. (2016). *De novo* sequencing and transcriptome analysis of *Pleurotus eryngii* subsp. *tuoliensis* (Bailinggu) mycelia in response to cold stimulation. Molecules 21, E560. 10.3390/molecules21050560 PMC6273410 27196889

[B14] Fu Y. P. Dai Y. T. Chethana K. W. T. Li Z. Sun L. Li C. (2022). Large-scale genome investigations reveal insights into domestication of cultivated mushrooms. Mycosphere 13, 86–133. 10.5943/mycosphere/si/1f/4

[B15] Gu J. F. Wei Q. Y. (2010). Resistance mechanisms of fungi and research progress on new antifungal medicine. Pharm. Clin. Res. 18, 303–306. 10.13664/j.cnki.pcr.2010.03.018

[B16] Hannuksela T. Hervé du Penhoat C. (2004). NMR structural determination of dissolved O-acetylated galactoglucomannan isolated from spruce thermomechanical pulp. Carbohydr. Res. 339, 301–312. 10.1016/j.carres.2003.10.025 14698888

[B17] Harris P. V. Welner D. McFarland K. C. Re E. Navarro Poulsen J. C. Brown K. (2010). Stimulation of lignocellulosic biomass hydrolysis by proteins of glycoside hydrolase family 61: structure and function of a large, enigmatic family. Biochemistry 49, 3305–3316. 10.1021/bi100009p 20230050

[B18] Hori C. Ishida T. Igarashi K. Samejima M. Suzuki H. Master E. (2014). Analysis of the *Phlebiopsis gigantea* genome, transcriptome and secretome provides insight into its pioneer colonization strategies of wood. PLoS Genet. 10, e1004759. 10.1371/journal.pgen.1004759 25474575 PMC4256170

[B19] Jiang N. Li Z. Dai Y. Liu Z. Han X. Li Y. (2022). Massive genome investigations reveal insights of prevalent introgression for environmental adaptation and triterpene biosynthesis in *Ganoderma* . Mol. Ecol. Resour. 10. 10.1111/1755-0998.13718 36214617

[B20] Katoh K. Standley D. M. (2013). MAFFT multiple sequence alignment software version 7: improvements in performance and usability. Mol. Biol. Evol. 30, 772–780. 10.1093/molbev/mst010 23329690 PMC3603318

[B21] Kaur G. Ganjewala D. Bist V. Verma P. C. (2019). Antifungal and larvicidal activities of two acyclic monoterpenes, citral and geraniol against phytopathogenic fungi and insects. Archives Phytopathology Plant Prot. 52, 458–469. 10.1080/03235408.2019.1651579

[B22] Kligun E. Ostretsov B. Titievsky A. Farkov M. Alamouti S. M. Brodsky L. (2017). Adaptation of the pine fungal pathogen *Grosmannia clavigera* to monoterpenes: biochemical mechanisms revealed by RNA‐seq analysis. For. Pathol. 47, e12372. 10.1111/efp.12372

[B23] Kubo Y. (2013). Function of peroxisomes in plant-pathogen interactions. Subcell. Biochem. 69, 329–345. 10.1007/978-94-007-6889-5_18 23821157

[B24] Li L. Stoeckert C. J. J. Roos D. S. (2003). OrthoMCL: identification of ortholog groups for eukaryotic genomes. Genome Res. 13, 2178–2189. 10.1101/gr.1224503 12952885 PMC403725

[B25] Li X. Xu Y. Y. Shen S. L. Yin X. Klee H. Zhang B. (2017). Transcription factor CitERF71 activates the terpene synthase gene CitTPS16 involved in the synthesis of E-geraniol in sweet orange fruit. J. Exp. Bot. 68, 4929–4938. 10.1093/jxb/erx316 28992329 PMC5853461

[B26] Li J. Han L. H. Liu X. B. Zhao Z. W. Yang Z. L. (2020). The saprotrophic *Pleurotus ostreatus* species complex: late Eocene origin in East Asia, multiple dispersal, and complex speciation. IMA Fungus 11, 10. 10.1186/s43008-020-00031-1 32617259 PMC7325090

[B62] Li F. Zhao Y. Xue L. Ma F. Y. Dai S. Y. Xie S. X. (2022). Microbial lignin valorization through depolymerization to aromatics conversion. Trends in biotechnology 40, 1469–1487. 10.1016/j.tibtech.2022.09.009 36307230

[B27] Lin C. P. Huang J. P. Wu C. S. Hsu C. Y. Chaw S. M. (2010). Comparative chloroplast genomics reveals the evolution of Pinaceae genera and subfamilies. Genome Biol. Evol. 2, 504–517. 10.1093/gbe/evq036 20651328 PMC2997556

[B28] Liu X. B. Li J. Horak E. Yang Z. L. (2016). *Pleurotus placentodes*, originally described from Sikkim, rediscovered after 164 years. Phytotaxa 267, 137–145. 10.11646/phytotaxa.267.2.6

[B29] Liu C. J. Duan Y. L. Jin R. Z. Han Y. Y. Hao J. H. Fan S. X. (2018). Spent mushroom substrates as component of growing media for lettuce seedlings. IOP Conf. Ser. Earth Environ. Sci. 185, 012016. 10.1088/1755-1315/185/1/012016

[B30] Liu H. L. Wu S. G. Li A. Ruan J. (2021). SMARTdenovo: a *de novo* assembler using long noisy reads. GigaByte 8, gigabyte15–9. 10.46471/gigabyte.15 PMC9632051 36824332

[B31] Parra G. Bradnam K. Korf I. (2007). CEGMA: a pipeline to accurately annotate core genes in eukaryotic genomes. Bioinformatics 23, 1061–1067. 10.1093/bioinformatics/btm071 17332020

[B32] Qi X. Z. Liu L. Wang J. (2019). RNA-Seq reveals changes of gene expression and cellular metabolism caused by exogenous oxidative stress (H_2_O_2_) in *Foc*4. Acta Microbiol. Sin. 59, 891–906. 10.13343/j.cnki.wsxb.20180341

[B33] Rai R. Kaur B. Singh S. Di Falco M. Tsang A. Chadha B. S. (2016). Evaluation of secretome of highly efficient lignocellulolytic *Penicillium* sp. Dal 5 isolated from rhizosphere of conifers. Bioresour. Technol. 216, 958–967. 10.1016/j.biortech.2016.06.040 27341464

[B34] Rollefson J. B. Stephen C. S. Tien M. Bond D. R. (2011). Identification of an extracellular polysaccharide network essential for cytochrome anchoring and biofilm formation in *Geobacter sulfurreducens* . J. Bacteriol. 193, 1023–1033. 10.1128/jb.01092-10 21169487 PMC3067572

[B35] Rominiyi O. Adaramola B. Ikumapayi O. Oginni O. T. Akinola S. A. (2017). Potential utilization of sawdust in energy, manufacturing and agricultural industry, waste to wealth. World J. Eng. Technol. 5, 526–539. 10.4236/wjet.2017.53045

[B36] Scariot F. J. Pansera M. S. Delamare A. P. L. Echeverrigaray S. (2021). Citral and geraniol induce necrotic and apoptotic cell death on *Saccharomyces cerevisiae* . World J. Microbiol. Biotechnol. 37, 42. 10.1007/s11274-021-03011-8 33547564

[B37] Silori G. K. Kushwaha N. Kumar V. (2019). Essential oils from pines: chemistry and applications. Essent. Oil Res., 275–297. 10.1007/978-3-030-16546-8_10

[B38] Sossah F. L. Liu Z. H. Yang C. T. Okorley B. A. Sun L. Fu Y. (2019). Genome sequencing of *Cladobotryum protrusum* provides insights into the evolution and pathogenic mechanisms of the cobweb disease pathogen on cultivated mushroom. Genes. 10, E124. 10.3390/genes10020124 PMC6409746 30744046

[B39] Stamatakis A. (2014). RAxML version 8: a tool for phylogenetic analysis and post-analysis of large phylogenies. Bioinformatics 30, 1312–1313. 10.1093/bioinformatics/btu033 24451623 PMC3998144

[B40] Tang H. Bowers J. E. Wang X. Ming R. Alam M. Paterson A. H. (2008). Synteny and collinearity in plant genomes. Science 320, 486–488. 10.1126/science.1153917 18436778

[B41] Tasseva G. Virville J. Cantrel C. Moreau F. Zachowski A. (2004). Changes in the endoplasmic reticulum lipid properties in response to low temperature in Brassica napus. Plant Physiology Biochem. 42, 811–822. 10.1016/j.plaphy.2004.10.001 15596101

[B42] Terrett O. M. Lyczakowski J. J. Yu L. Iuga D. Franks W. T. Brown S. P. (2019). Molecular architecture of softwood revealed by solid-state NMR. Nat. Commun. 10, 4978. 10.1038/s41467-019-12979-9 31673042 PMC6823442

[B43] Wang L. Y. Li S. T. Guo L. H. Jiang R. (2003). Cloning and identification of the priming glycosyltransferase gene involved in exopolysaccharide 139A biosynthesis in Streptomyces. Acta Genet. Sin. 30, 723–729. 14682240

[B44] Wang Y. Lim L. DiGuistini S. Robertson G. Bohlmann J. Breuil C. (2013). A specialized ABC efflux transporter GcABC‐G 1 confers monoterpene resistance to *Grosmannia clavigera*, a bark beetle‐associated fungal pathogen of pine trees. New Phytol. 197, 886–898. 10.1111/nph.12063 23252416

[B45] Wang H. J. Li G. J. Zhao R. L. Nakagawa K. Kimura F. Miyazawa T. (2018). Artificial cultivation and nutrition analysis of *Pleurotus placentodes* wild strain. Mycosystema 37, 606–613. 10.1002/jbm.a.36252

[B46] Wang X. Zhang Y. Zhang R. Zhang J. (2019a). The diversity of pattern recognition receptors (PRRs) involved with insect defense against pathogens. Curr. Opin. Insect Sci. 33, 105–110. 10.1016/j.cois.2019.05.004 31358188

[B47] Wang X. X. Peng J. Y. Sun L. Bonito G. Wang J. Cui W. (2019b). Genome sequencing illustrates the genetic basis of the pharmacological properties of *Gloeostereum incarnatum* . Genes. 10, E188. 10.3390/genes10030188 PMC6470497 30832255

[B48] Wang Q. Wang X. F. Yang X. J. Guo Y. F. Zhou T. (2021). Effects of culture substrates on nutritional and flavor components of *Pleurotus pulmonarius* . Mycosystema 40, 3182–3195. 10.13346/j.mycosystema.210257

[B49] Wang M. Xu H. Liu C. Tao Y. Wang X. Liang Y. (2022). Peroxisome proliferator FpPEX11 is involved in the development and pathogenicity in *Fusarium pseudograminearum* . Int. J. Mol. Sci. 23, 12184. 10.3390/ijms232012184 36293041 PMC9603656

[B50] Waterhouse R. M. Seppey M. Simao F. A. Manni M. Ioannidis P. Klioutchnikov G. (2018). BUSCO applications from quality assessments to gene prediction and phylogenomics. Mol. Biol. Evol. 35, 543–548. 10.1093/molbev/msx319 29220515 PMC5850278

[B51] Weijn A. Bastiaan-Net S. Wichers H. J. Mes J. J. (2013). Melanin biosynthesis pathway in *Agaricus bisporus* mushrooms. Fungal Genet. Biol. 55, 42–53. 10.1016/j.fgb.2012.10.004 23123422

[B52] Wu H. Nakazawa T. Xu H. Yang R. Bao D. Kawauchi M. (2021). Comparative transcriptional analyses of *Pleurotus ostreatus* mutants on beech wood and rice straw shed light on substrate-biased gene regulation. Appl. Microbiol. Biotechnol. 105, 1175–1190. 10.1007/s00253-020-11087-9 33415371

[B53] Xie H. Zhou Y. A. Dou J. Ren M. G. Zhang Q. B. Ma Y. X. (2011). Preliminary study on efflux pump gene and fluconazole resistance in *Candida Tropicalis* . Chin. J. Clin. Electron. Ed. 5, 7395–7398. 10.3877/cma.j.issn.1674-0785.2011.24.046

[B54] Xu C. X. Ruan H. Q. Cai W. J. Staehelin C. Dai W. (2021a). Identification of an exopolysaccharide biosynthesis gene in *Bradyrhizobium diazoefficiens* USDA110. Microorganisms 9, 2490. 10.3390/microorganisms9122490 34946092 PMC8707904

[B55] Xu F. Chen P. Li H. Qiao S. Wang J. Wang Y. (2021b). Comparative transcriptome analysis reveals the differential response to cadmium stress of two *Pleurotus* fungi: *pleurotus cornucopiae* and *Pleurotus ostreatus* . J. Hazard. Mater. 416, 125814. 10.1016/j.jhazmat.2021.125814 33866290

[B56] Yang X. Y. Zeng Z. H. Yan J. Y. Fan W. Bian H. W. Zhu M. Y. (2013). Association of specific pectin methylesterases with Al-induced root elongation inhibition in rice. Physiol. Plant. 148, 502–511. 10.1111/ppl.12005 23136980

[B57] Yao S. Liu Y. Y. Zhao C. Yan J. J. Xie B. G. (2019). Unsaturated fatty acids as potential regulator involved in stipe elongation of *Flammulina filiformis* . Mycosystema 038, 2232–2240. 10.13346/j.mycosystema.190269

[B58] Yuan Z. Chen L. (2014). The role of endophytic fungal individuals and communities in the decomposition of *Pinus massoniana* needle litter. PLoS One 9, e105911. 10.1371/journal.pone.0105911 25157631 PMC4144953

[B59] Zhang Y. H. P. Himmel M. E. Mielenz J. R. (2006). Outlook for cellulase improvement: screening and selection strategies. Biotechnol. Adv. 24, 452–481. 10.1016/j.biotechadv.2006.03.003 16690241

[B60] Zhang G. Yan P. Leng D. Shang L. Zhang C. Wu Z. (2022). Functional roles of LaeA-like genes in fungal growth, cellulase activity, and secondary metabolism in *Pleurotus ostreatus* . J. Fungi 8, 902. 10.3390/jof8090902 PMC9502681 36135627

[B61] Zhang F. J. (2015). Functional analysis of AMPK/Cnrk1 in the transcription regulation of cellulase genes in *Trichoderma reesei* . Shandong University. 10.7666/d.Y2793335

