# Supplementary figures and images for: Comparative genomic analysis of pleurotus species reveals insights into the evolution and coniferous utilization of Pleurotus placentodes
Source: Front Mol Biosci. 2023 Nov 6;10:1292556. doi: 10.3389/fmolb.2023.1292556 (PMC10658006; doi:10.3389/fmolb.2023.1292556)

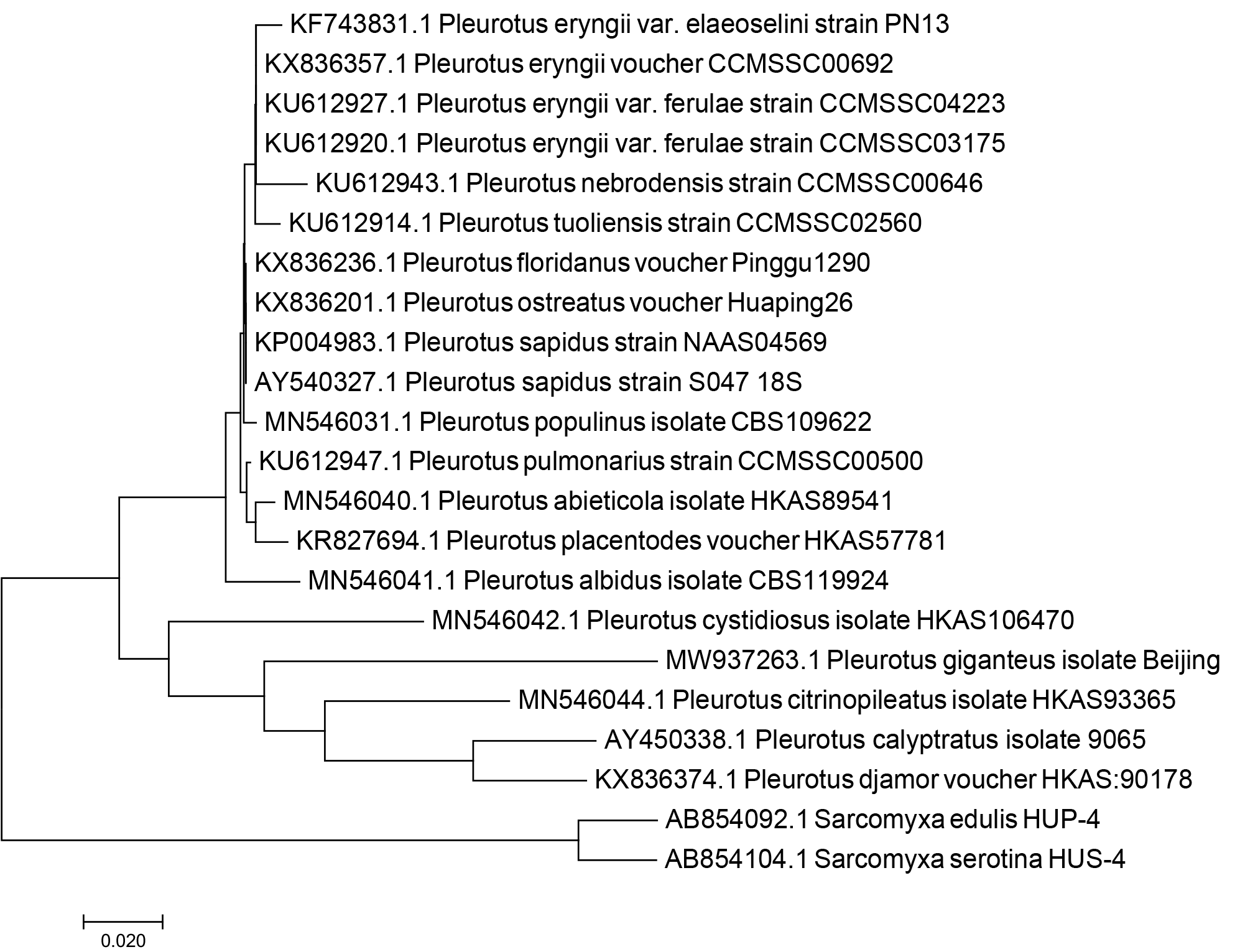

Supplement: Supplementary file 3 [file Image1.TIF]
